# High frequency stimulation activates hot spots of spontaneous synaptic transmission

**DOI:** 10.3389/fnsyn.2025.1539868

**Published:** 2025-04-14

**Authors:** Herson Astacio, Maria Bykhovskaia

**Affiliations:** Department of Neurology, Wayne State University, Detroit, MI, United States

**Keywords:** active zone, GCaMP, high frequency stimulation, *Drosophila*, neuromuscular junction

## Abstract

Neuronal transmitters are released at the morphological specializations known as active zones (AZs). Transmitters can be released either in response to a stimulus or spontaneously, and spontaneous transmission is a vital component of neuronal communication. Employing postsynaptically tethered calcium sensor GCaMP, we investigated how nerve stimulation affects spontaneous transmission at individual AZs at the *Drosophila* neuromuscular synapse. Optical monitoring of spontaneous transmission at individual AZs revealed that prolonged high-frequency stimulation (HFS, 30 Hz for 1 min) selectively activates the hot spots of spontaneous transmission, including the individual AZs with elevated activities as well as AZ clusters. In contrast, a brief tetanus (2 s) activated numerous low-activity AZs. We employed Monte-Carlo simulations of spontaneous transmission based on a three-state model of AZ preparedness, which incorporated longer-lasting (minutes) and shorter-lasting (sub-seconds to seconds) high-activity states of AZs. The simulations produced an accurate quantitative description of the variability and time-course of spontaneous transmission at individual AZs before and after the stimulation and suggested that HFS activates both longer-lasting and shorter-lasting states of AZ preparedness.

## 1 Introduction

Neuronal transmitters are released at morphological specializations termed active zones (AZs), which represent clusters of voltage gated Ca^2+^ channels (VGCCs) (Dolphin and Lee, 2020) surrounded by synaptic vesicles (SVs) and organized by scaffolding proteins (Gramlich and Klyachko, 2019; Ghelani and Sigrist, 2018). An action potential triggers Ca^2+^ influx, which in turn drives the fusion of SVs with the presynaptic membrane and release of neuronal transmitters into the synaptic cleft. SV fusion can also occur in a spontaneous mode, which is not directly coupled to the Ca^2+^ influx but may depend on local Ca^2+^ transients (Kavalali, 2019; Reese and Kavalali, 2015; Llano et al., 2000; Williams et al., 2012; Lefkowitz et al., 2009; Collin et al., 2005; Simkus and Stricker, 2002; Williams and Smith, 2018).

Early studies considered spontaneous release as a “leak” from evoked transmission (Fatt and Katz, 1952; Kaeser and Regehr, 2014), however, more recently it was demonstrated that the evoked and spontaneous release components have distinctions (Kaeser and Regehr, 2014; Chanaday and Kavalali, 2018; Kavalali, 2015). Although spontaneous events show some dependence on Ca^2+^ (Williams and Smith, 2018; Kavalali, 2019; Schneggenburger and Rosenmund, 2015), including VGCCs (Williams et al., 2012; Ermolyuk et al., 2013; Goswami et al., 2012) and internal Ca^2+^ stores (Llano et al., 2000; Emptage et al., 2001), the Ca^2+^ dependence of the spontaneous release is very weak, in contrast to the evoked release (Williams and Smith, 2018; Kochubey and Schneggenburger, 2011; Sun et al., 2007; Borst and Sakmann, 1996), and a Ca^2+^-independent population of spontaneous events has been identified at several preparations (Kochubey and Schneggenburger, 2011; Vyleta and Smith, 2011; Abenavoli et al., 2002; Lou et al., 2005; Hua et al., 1998). Furthermore, the SV functional pools controlling the evoked and spontaneous release are at least partially segregated (Schneggenburger and Rosenmund, 2015; Crawford and Kavalali, 2015; Hablitz et al., 2009; Koenig and Ikeda, 1999; Fredj and Burrone, 2009; Sara et al., 2005; but see also Hua et al., 2010; Wilhelm et al., 2010) and have distinctions in the fusion machinery.

Recent optical studies in *Drosophila* suggested that a sub-population of AZs may be selectively tuned for the spontaneous transmission. These studies generated *Drosophila* lines that expressed the Ca^2+^ sensor GCaMP tethered to postsynaptic specializations, and this approach enabled the detection of highly localized postsynaptic responses (Peled et al., 2014; Melom et al., 2013). Super-resolution microscopy coupled with electrophysiology convincingly demonstrated that this technique enables the detection of single fusion events at individual AZs (Astacio et al., 2022; Akbergenova et al., 2018; Newman et al., 2022). This approach revealed that different subsets of AZs usually show elevated activity for either evoked or spontaneous transmission, but typically not both, even though numerous AZs were capable of generating both release modes with low probabilities(Melom et al., 2013). Furthermore, the evoked and spontaneous release components were totally segregated and even negatively correlated in a mutant with distorted AZs (Peled et al., 2014). In contrast, a weak (but statistically significant) positive correlation was detected at well-defined AZs of wild type (WT) larvae (Grasskamp et al., 2023). Interestingly, the latter study also identified a sub-population of AZs as “spontaneous only.” To reconcile the apparent controversy, the authors proposed that a sub-population of AZs represent a “mixed channel” with both evoked and spontaneous transmission modes governed by similar fusion machinery, while other AZs represent a “dedicated spontaneous” communication channel (Grasskamp et al., 2023). The dedicated spontaneous communication channel likely represents a distinct form of neuronal communication (Kavalali, 2018), which regulates neuronal and behavioral plasticity, neuronal development, and homeostasis (Kavalali, 2015; Sutton et al., 2006; McKinney et al., 1999; Choi et al., 2014) and utilizes a dedicated sub-population of AZs (Peled et al., 2014; Melom et al., 2013).

Electrophysiology studies showed that the timings of spontaneous release events are not random (Abenavoli et al., 2002; Cohen et al., 1974; Cohen et al., 1981; Leao et al., 2005), suggesting heterogeneity in spontaneous transmission. A recent study demonstrated the heterogeneity in spontaneous transmission directly by coupling electrophysiology and optical detection of release events at individual AZs (Astacio et al., 2022). The latter study discovered that the spontaneous events represent a mixture of two populations: (1) those with random variations described by the Poissonian low, and (2) the “hot spots” with elevated release probabilities, likely representing the dedicated communication channel (Grasskamp et al., 2023).

Spontaneous transmission can be enhanced by high-frequency stimulation (HFS) (Yoshihara et al., 2005; Vandael et al., 2020; Vandael and Jonas, 2024; Lin et al., 2020), and it was shown that this mechanism depends on the retrograde signaling pathway (Yoshihara et al., 2005) and the presynaptic fusion machinery (Lin et al., 2020; Cho et al., 2015). Since the locally enhanced spontaneous activity could generate a positive feedback loop for the activity-dependent synaptic restructuring (Choi et al., 2014; Yoshihara et al., 2005; Cho et al., 2015; Harris and Littleton, 2015), we questioned whether HFS promotes the hot spots and heterogeneity in spontaneous transmission. We took advantage of transgenic Drosophila expressing the Ca^2+^ sensor GCaMP5 tethered to the postsynaptic reticulum (Melom et al., 2013), which allows optical detection of single spontaneous fusion events at the neuromuscular junction (NMJ), and investigated how HFS affects spontaneous transmission at individual AZs.

## 2 Materials and methods

*Drosophila melanogaster* were cultured on standard medium at 25°C. The line expressing myrGCaMP5 (Melom et al., 2013) was used for optical detection of spontaneous events. Third instar larvae were dissected in the modified HL3 solution containing (in mM): 70 NaCl, 5 KCl, 20 MgCl_2_, 10 NaHCO_3_, 5 trehalose, 115 sucrose, 2.5 HEPES Acid, 2.5 HEPES Salt, and 1 CaCl_2_ (Melom et al., 2013). All the experiments were performed on the Ib type boutons of the muscles 6 and 7 at abdominal segments 2–4.

Videorecordings of GCaMP-expressing NMJs were performed continuously at 25 frames per second. The recording setup was built around Nikon Eclipse FN-1 upright microscope equipped with epifluorescence (Omega Optical XF115-2 FITC longpass filter set), x60 water immersion objective (2.8 mm working distance, 1.0 NA), X-CITE 120 LED lamp, and sCMOS PCO edge camera.

The nerve stimulation at a frequency of 10 or 30 Hz was performed via the suction electrode with Master-8 pulse stimulator (AMPI, Jerusalem, Israel). Although it was shown that the prolonged stimulation at the frequencies of 10 or 30 Hz is not damaging to our preparation (Akbergenova and Bykhovskaia, 2007; Vasin et al., 2014), we visually monitored the evoked GCaMP activity during the stimulation to ensure that it was steady over the entire tetanus and reduced to the background level once the stimulation was terminated. The post HFS recordings of spontaneous transmission were started in 30 s upon the termination of the stimulation.

The experiments which showed any signs of nerve damage during the stimulation, such as a lack of muscle contraction or relaxation, or disappearance of the evoked GCaMP signal, were discarded. In addition, in each experiment we ensured that the spontaneous activity over the entire recording period remained at a steady level and was clearly detectable, including the recordings before and after HFS. The experiments which showed a run-down of the spontaneous GCaMP signal or an out-of-plain deviation or distortion of the analyzed NMJ were discarded.

**Figure 1:**
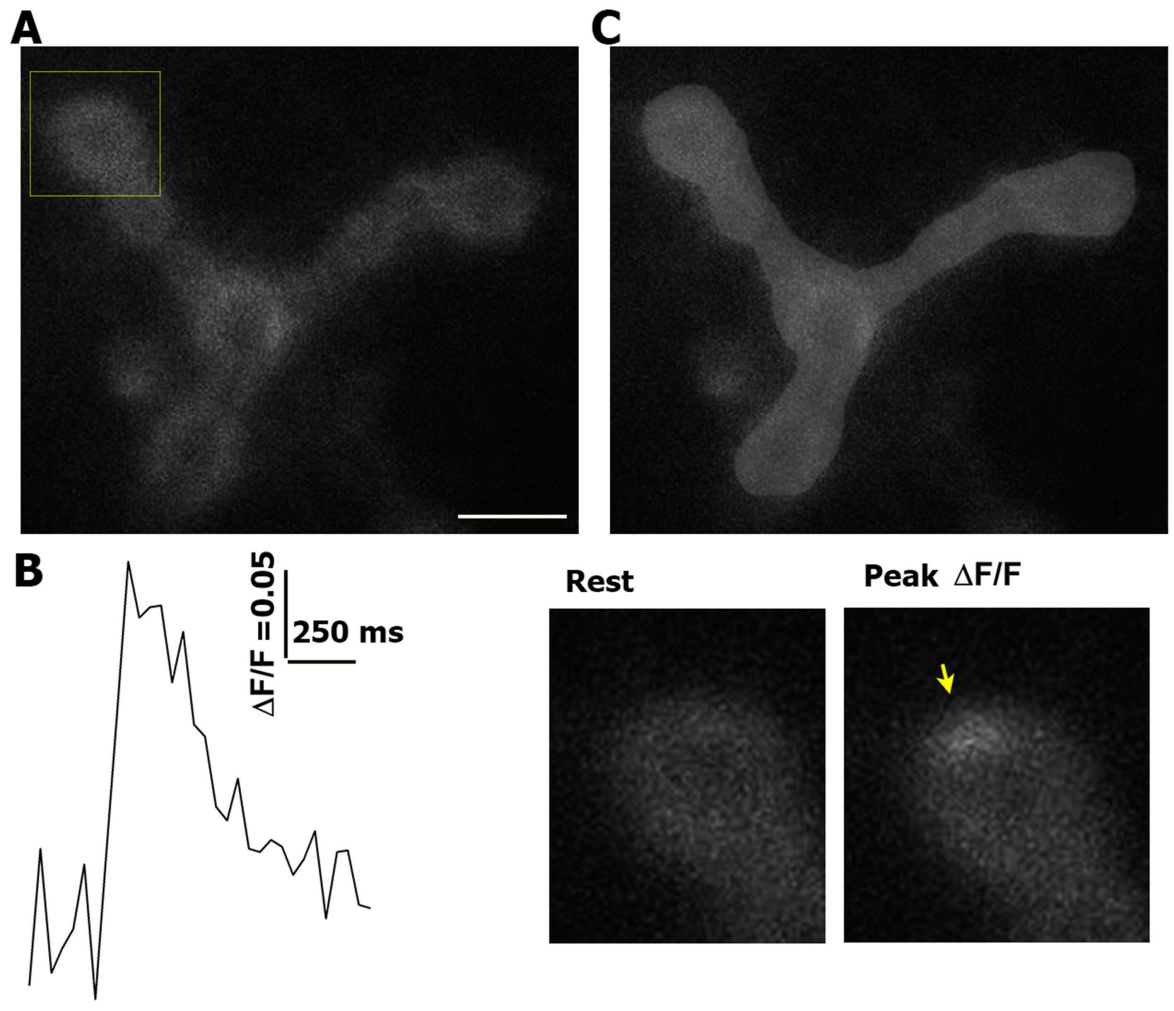
Event detection and area measurements. **(A)** An example of an NMJ expressing GCaMP5. The square marks the bouton enlarged in panel **(B)**. Scale bar: 5 μm. **(B)** An example of a detected event. An arrow marks the event at the peak of GCaMP5 signal. **(C)** The outlined area of the NMJ (light gray).

To generate fluorescence profiles of optical events (Figures 1A,B), we used ImageJ software (National Institute of Health). The NMJ area was outlined manually (Figure 1C) with a standard brush (10 pixel diameter). The same area was used to analyze the pre- and post-HFS recordings, and only the outlined area of the NMJ was included in the event analysis. We outlined and included only the parts of the NMJ that were in one plain and showed clear GCaMP signal with no distortions.

The in-house software package (Astacio et al., 2022) was used for the semi-automatic detection of optical events and the Poissonian analysis of the activity distributions. Briefly, the software package included three levels: (1) event detection; (2) AZ sorting; and (3) statistical analysis. At the first level, the fluorescent puncta at each frame were detected employing the amplitude (ΔF/*F* > 0.5) and area (>5 pixels) thresholds. At the second level, hierarchical cluster analysis was performed to classify and assign the detected events, generating the ensemble of AZs. The cutoff of 3 pixels (0.324 μm) between the weighted centroids of the detected puncta served as a threshold for assigning the events to a single AZ. The verification of this algorithm with super-resolution microscopy (Astacio et al., 2022) showed that the detected AZs match those determined using the AZ marked Bruchpilot, with only a small proportion of AZs separated by <0.3 μm being lost.

The Poissonian analysis employed a computational algorithm to evaluate the number of AZs deviating from the Poissonian law. The algorithm sequentially eliminated high-activity (HA) AZs one by one; after each elimination, the remaining ensemble was tested for the Poissonian fit. Latency analysis, Monte-Carlo (MC) simulations, and investigating the clusters of activity were performed as described in Astacio et al. (2022).

## 3 Results

**Figure 2:**
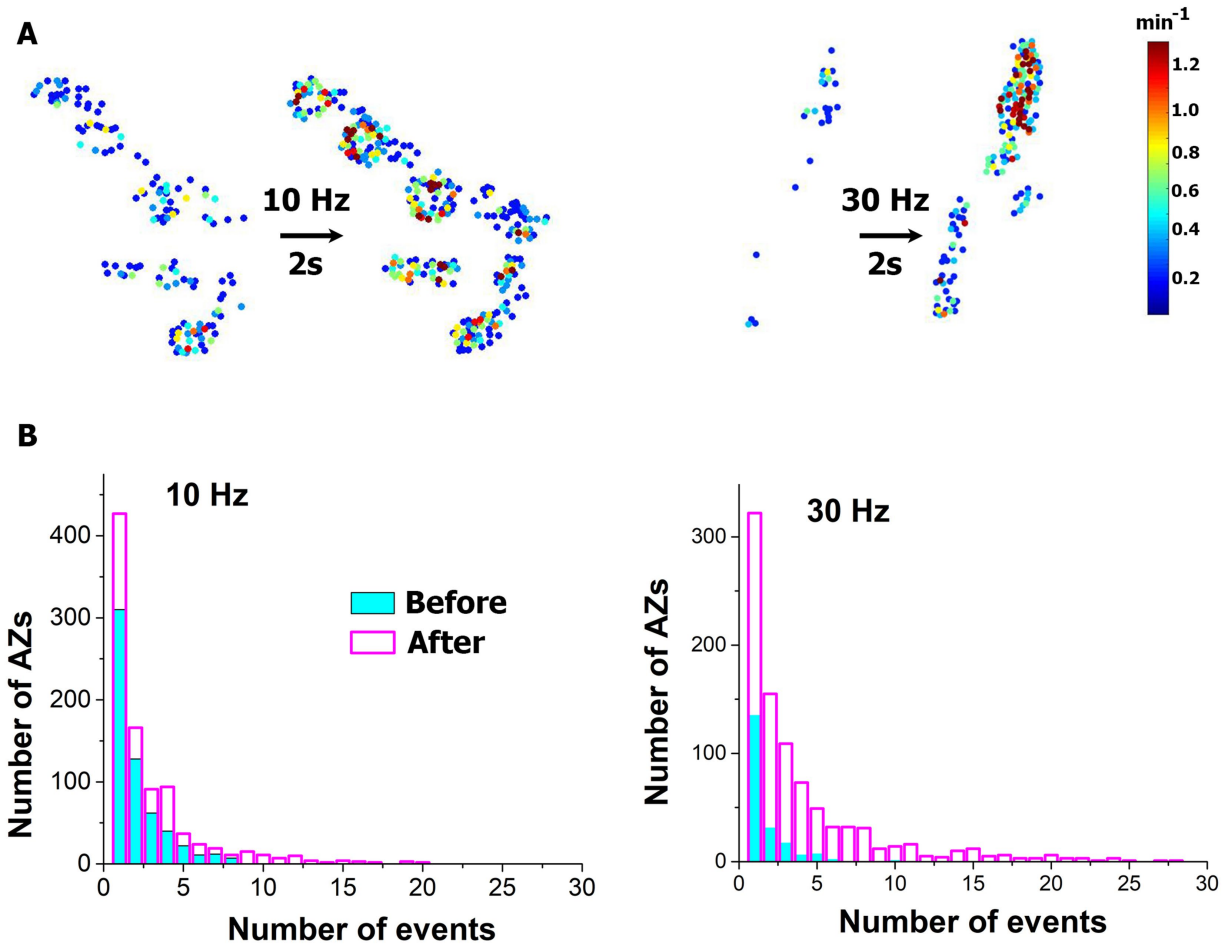
Brief HFS tetanus broadly enhances spontaneous activity across AZ ensemble. **(A)** Heatmaps of representative NMJs showing increased numbers of responding AZs and enhanced activities at individual AZs upon the tetanus. **(B)** Distributions of activities across AZs before (cyan) and after (magenta) the tetanus. Note the increased numbers of AZs producing spontaneous events. The plots show the pooled data collected from six NMJ at each condition (either 10 or 30 Hz stimulation frequency).

We first investigated how a brief HFS tetanus (2 s at either 10 or 30 Hz stimulation frequency) affects spontaneous transmission at individual AZs. Spontaneous transmission was recorded for 5 min, then the 2 s tetanus was applied, and then the recording was repeated. In agreement with (Cho et al., 2015), we observed a broad increase in activities within the entire ensemble of AZs (Figures 2A,B), with numerous new AZs being activated. This increase in activities was more prominent for the 30 Hz tetanus.

**Figure 3:**
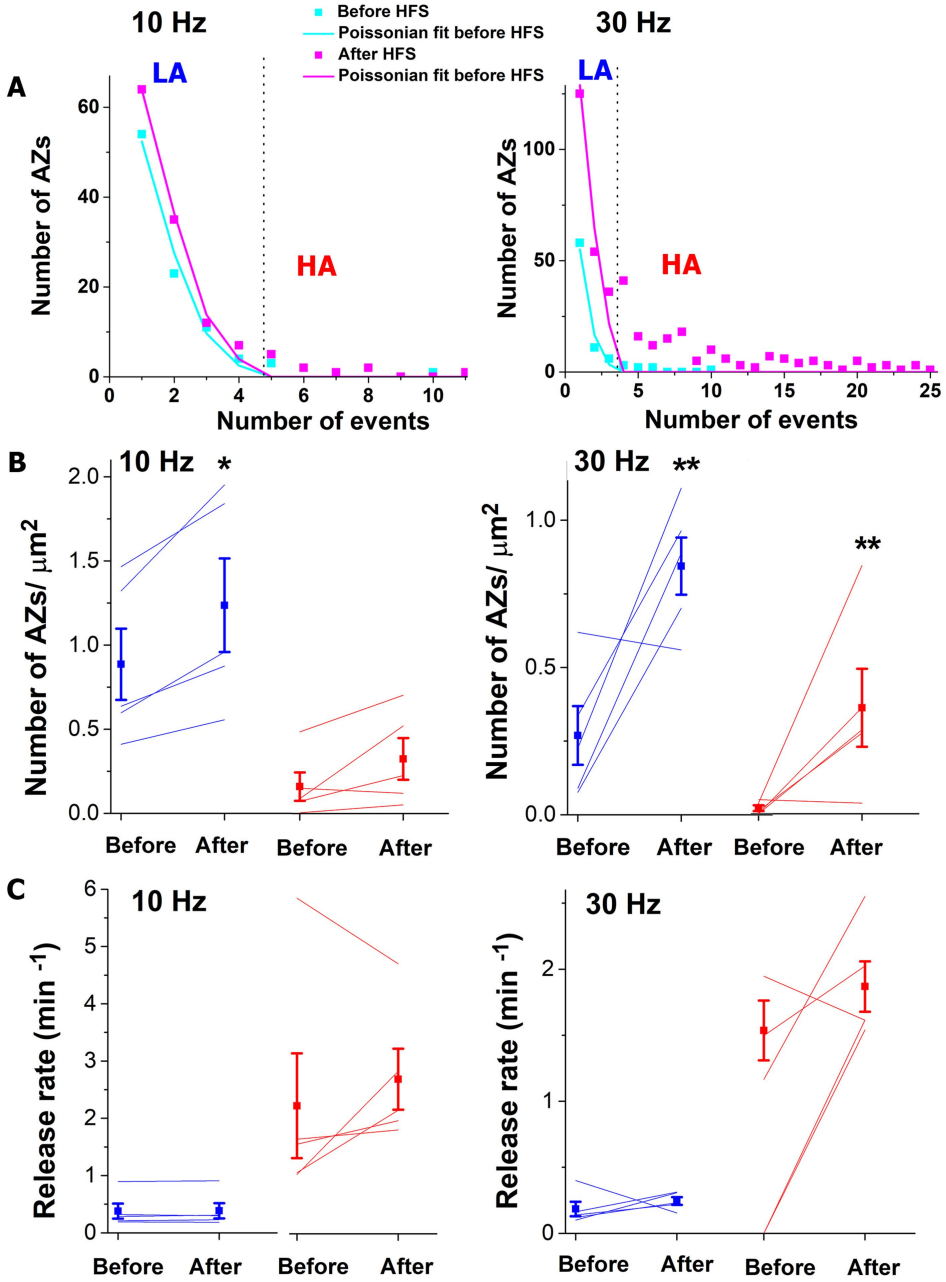
The brief HFS tetanus (2 s) recruits additional AZs into spontaneous transmission. **(A)** AZ sorting at individual experiments employing the Poissonian fit. The representative examples at 10 and 30 Hz are shown. AZs that fall within the Poissonian ensemble are marked as LA, while the remaining AZs are marked as HA. The dotted vertical lines mark the separation threshold obtained based on the Poissonian fit. **(B)** The 10 Hz tetanus produces a significant increase (*p* < 0.05) in the responding number of AZs classified as LA (blue) but not in those classified as HA (red). In contrast, the 30 Hz tetanus produces a significant increase in the responding number of AZs classified as HA (red, *p* < 0.01) as well as LA (blue). Thin lines correspond to individual experiments. **(C)** No significant change is observed in the release rate at individual AZs upon HFS. Blue: AZs classified as LA; red: AZs classified as HA.

We next identified the low-activity (LA) states of AZs belonging to the Poissonian ensemble and HA states of AZs that deviated from the Poissonian distribution (Astacio et al., 2022) (Figure 3A). Both 10 and 30 Hz tetani produced a significant increase in the number of AZs classified as LA (Figure 3B blue), and this increase was more prominent (approximately six-fold) for the 30 Hz tetanus. In addition, the 30 Hz tetanus also produced a significant (approximately 39-fold) increase in the number of AZs classified as HA (Figure 3B, 30 Hz, red). This increase in the number of AZs within the HA ensemble was not significant for the 10 Hz tetanus although a similar trend was observed. Interestingly, the activities of individual AZs in either group did not show a significant increase (Figure 3C).

Thus, the most evident effect of a short (2 s) HFS tetanus was a significant increase in the number of AZs producing spontaneous events. At a lower stimulation frequency (10 Hz) this effect was only significant for the LA ensemble, while a higher frequency (30 Hz) also induced a very prominent increase in the number of AZs within the HA ensemble, so that the proportion of AZs in their HA state significantly increased (Figure 3B). This result suggests that HFS can activate the AZs which were silent of nearly silent for spontaneous transmission, and also can convert AZs from their LA states to the HA states. We next questioned whether a more prolonged HFS would enhance either of these functions.

**Figure 4:**
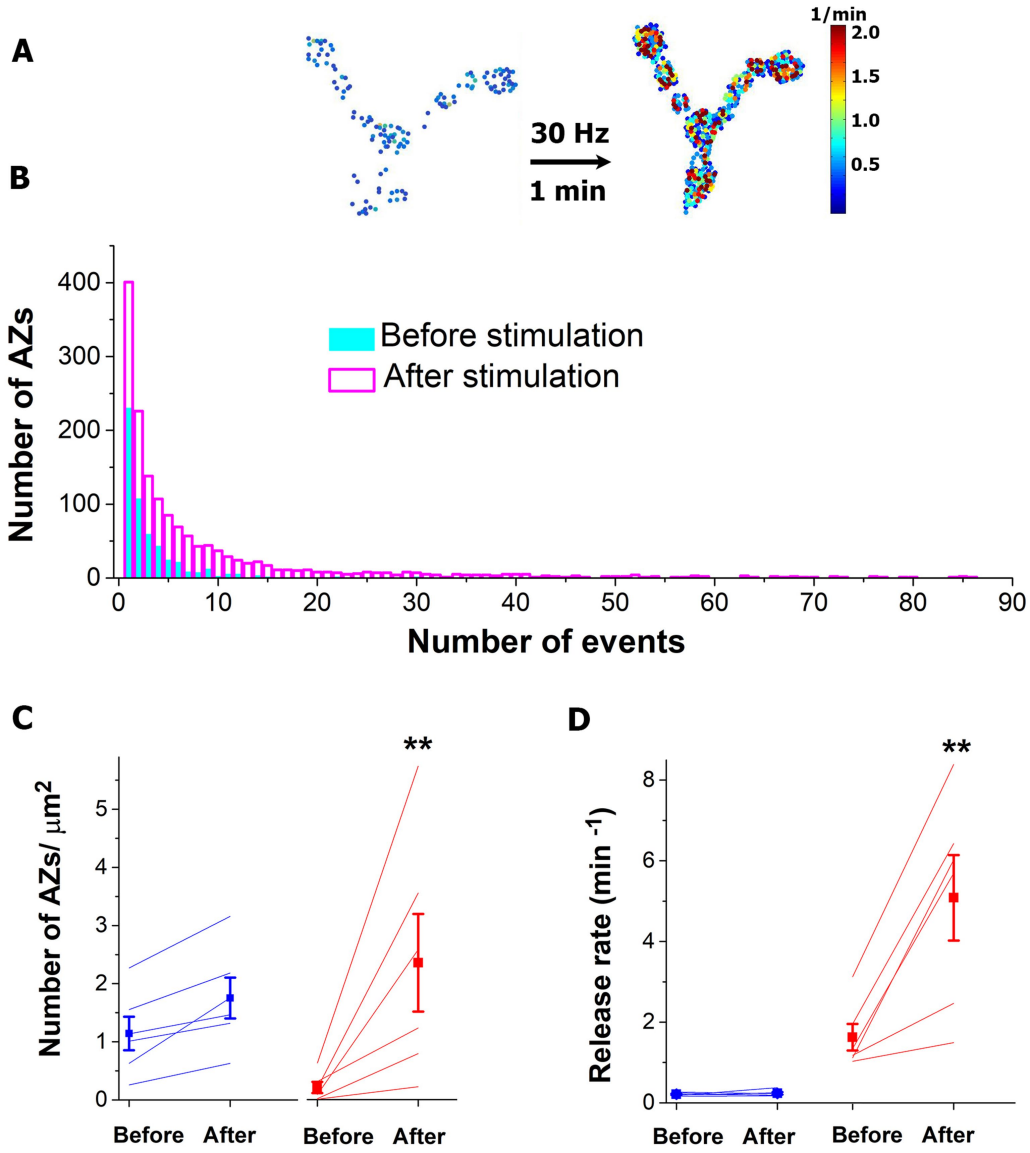
The prolonged HFS tetanus (1 min at 30 Hz) selectively promotes the HA states of AZs. **(A)** The heatmap of a representative NMJ showing numerous AZs in their HA states (red) activated by the HFS. **(B)** The activity distributions across AZ ensemble before and after the HFS tetanus. The plot shows the data pooled from six experiments. Note that multiple AZs started generating tens of events over the recording period upon HFS. **(C)** The number of AZs in their HA states (red) is selectively increased upon HFS (p < 0.01). **(D)** The release rates of AZs in their HA states are selectively increased upon HFS (*p* < 0.01). Blue lines correspond to AZs in their LA states.

The protocol described above was employed with a more prolonged HFS tetanus: 30 Hz for 1 min (Figures 4A,B). Notably, we observed a drastic increase in the number of AZs in their HA states (Figures 4A,C red). Interestingly, the number of AZs in their LA states did not show a significant increase (Figure 4C blue). This result suggests that during a prolonged HFS a conversion of AZs to their HA states may occur. Consistently, activities of the individual AZs in their HA states (but not in LA states, Figure 4D) significantly increased.

**Figure 5:**
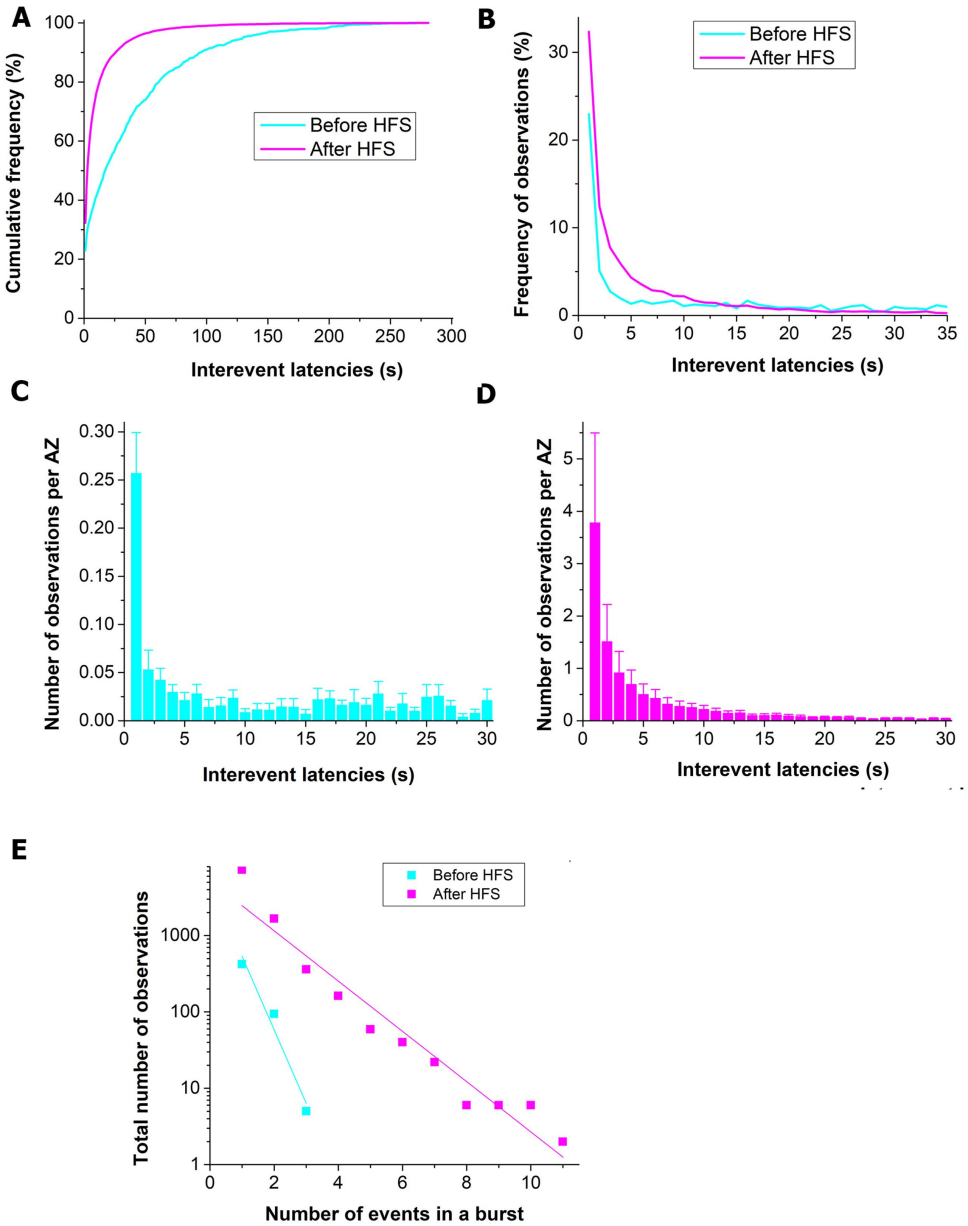
The prolonged HFS tetanus (1 min at 30 Hz) promotes spontaneous release events following each other with short inter-event latencies. **(A)** The cumulative frequency distribution is shifted toward shorter inter-event latencies for the post-HFS dataset (*p* < 0.001 per K.S. test). **(B)** The frequency distribution shows a relative increase in the number of spontaneous events with inter-event latencies of 30 s or shorter. **(C)** Before HFS, a pronounced peak is observed for the events following each other with an interval of 1 s or shorter. The bars show Mean ± S.E. (*n* = 6 NMJs) observations normalized by the number of AZs. **(D)** Upon HFS, in addition to the pronounced peak at the 1 s interval, numerous events with latencies of 2–10 s are observed. **(E)** HSF significantly increases the length of sequences of events that are released with sub-second intervals (the exponential decay b = −0.96 ± 0.17 before HFS vs. b = −0.33 ± 0.03 after HFS, *p* < 0.05).

We next examined how the prolonged HFS affects the latencies between successive events from single AZs. The distribution of inter-event latencies was significantly altered following HFS (1 min at 30 Hz, Figure 5A). More specifically, the number of events that followed each other with an interval of <10 s was selectively increased (Figure 5B). In agreement with an earlier study (Astacio et al., 2022), we observed a disproportionally large peak for the inter-event latencies of 1 s or less (Figure 5C) for the unstimulated dataset. Following HFS, the 1 s peak was still prominent, although a high occurrence was observed for the pairs of events with inter-event intervals of 2–5 s. We next examined the length of the sequences or bursts of the events that followed each other at sub-second internals. Notably, we found that upon HFS stimulated AZs were producing long bursts of events, while unstimulated AZs were capable of producing only two or three subsequent events (Figure 5E).

**Figure 6:**
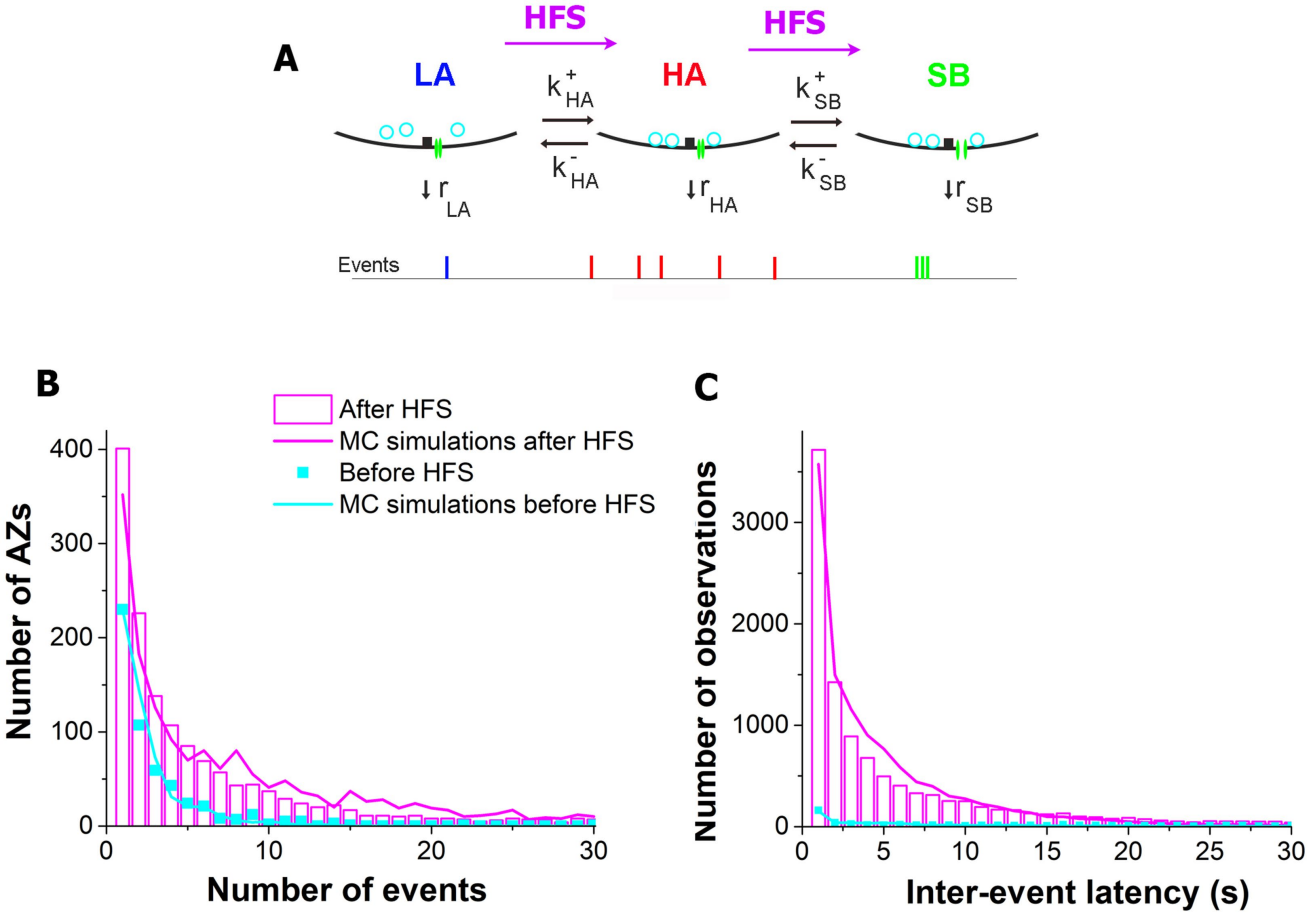
The three-state model provides excellent fit for the post-HFS experimental dataset when the parameters of the model are adjusted to promote the HA and SB states. **(A)** The three-state model (Astacio et al., 2022). An AZ can reside in the state of low activity LA, transition to the state of high activity HA, and subsequently transition to the state enabling sub-second bursts of events, SB. Each state is characterized by its own rate of release (Table 1). **(B)** The MC simulations performed based on the three-state model provide excellent fit for the activity distributions observed across AZs after (magenta) and before (cyan) HFS. The experimental data is pooled together for all the six experiments. **(C)** The MC simulations provide excellent fit for the latency distributions observed across AZs (the simulation parameters are identical for the outputs presented in the panels **B,C**).

To understand the biophysical processes underlying the activation of AZs upon HFS, we employed the model (Astacio et al., 2022) which incorporated the three states of activity for each AZ (Figure 6A), including the LA states corresponding to the Poissonian ensemble, the HA states, and the states enabling sub-second bursts (SB) of events. The parameters of the model (Figure 6A and Table 1) included the transitions between the states and the release rates at each of the states. Since MC simulations based on this model produced an excellent fit for the distributions of latencies and activities at individual AZs at rest (Astacio et al., 2022), we tested whether the model could accurately describe the post-HFS dataset (1 min at 30 Hz). We performed the MC simulations for the post-HFS and pre-HFS datasets, with the initial set of parameters for both datasets being taken from the simulations of the spontaneous activity at rest (Astacio et al., 2022). Subsequently, the parameters for the post-HFS dataset were optimized to produce the fit for the observed distributions of activities (Figure 6B) and latencies (Figure 6C) at individual AZs. Notably, the MC simulations produced an excellent fit for the post-HFS dataset, and only four parameters had to be modified (Table 1): (1) the total number of responding AZs (*N_AZ_*) was increased; (2) the transition to the HA state was promoted (by increasing *k^+^_HA_* and decreasing *k^−^_HA_*); and (3) the transition to the SB state was accelerated (by increasing in *k^+^_SB_*). Interestingly, the properties of the three functional states, i.e., their rates of release, did not require any modifications to fit the post-HFS dataset. These results suggest that HFS recruits extra AZs in spontaneous transmission and also promotes the transitions of AZs to the states of enhanced activities.

**Table 1:** Parameters describing the functional states of AZs and transitions between the states before and after HFS.

	*N_AZ_*	*r_LA_*	*k^+^_HA_*	*k^−^_HA_*	*r_HA_*	*k^+^_SB_*	*k^−^_SB_*	*r_SB_*
		*min ^−1^*						
Before	800	0.176	0.115	3.000	0.50	16.6	187.5	65.2
After	2000		0.200	1.500		37.5		

**Figure 7:**
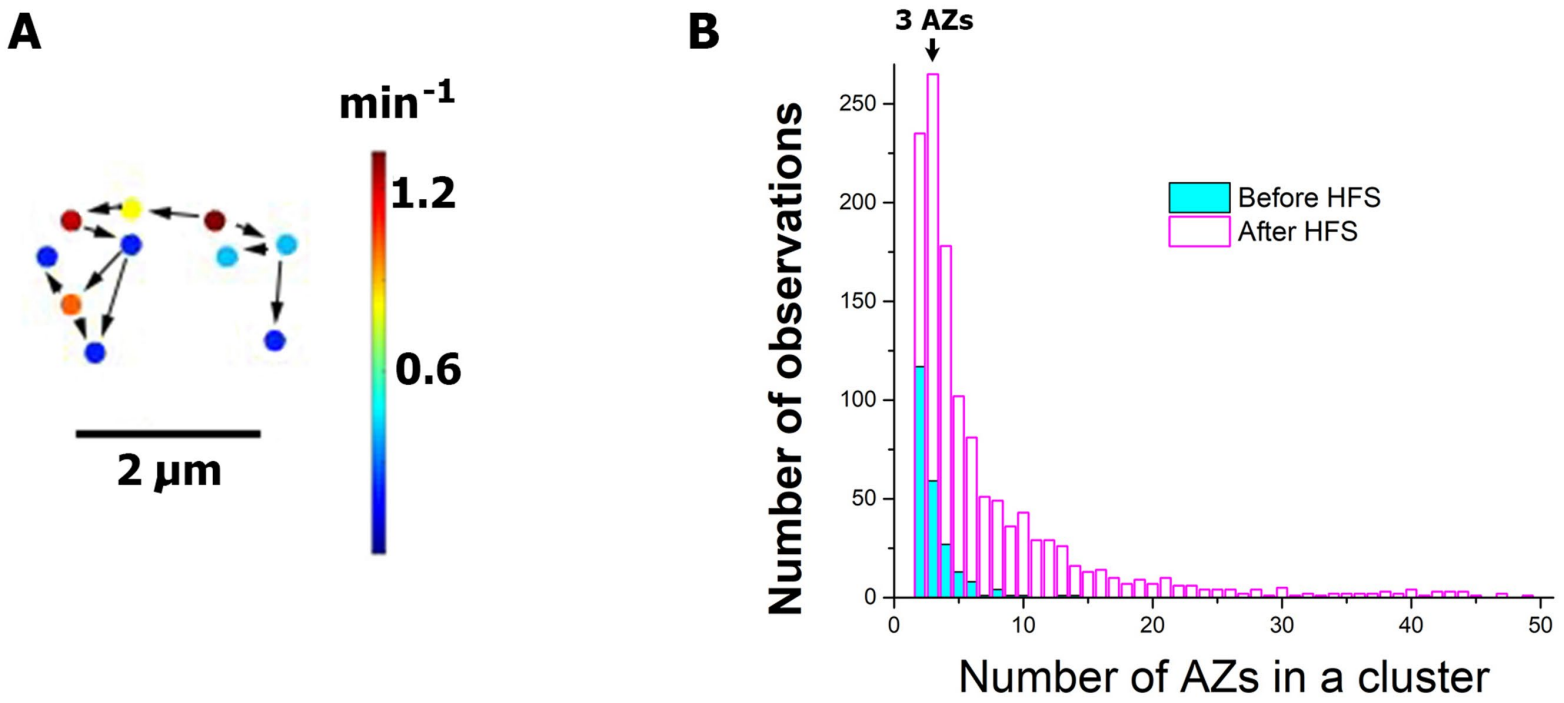
HFS activates the clusters of AZs with elevated spontaneous activities. **(A)** An example of an active cluster of AZs. Arrows connect neighboring AZs which generated sequential events following each other within a second. **(B)** The frequency distribution for the number of events in a cluster demonstrates that HFS (30 Hz for 1 min) significantly (*p* < 0.0001 per K.S. test) promotes the activity clusters. Note that at the resting NMJs (cyan) the majority of clusters have only two AZs, and not more than 10 AZs in a cluster are observed. In contrast, the stimulated NMJs (magenta) show the distribution mode at three AZs per cluster, and over 50 AZs are sometimes observed in a cluster, which may incorporate all the AZs within a bouton. The distribution shows the data pooled from six experiments.

Finally, we tested whether HFS activates AZ clusters, since our earlier study (Astacio et al., 2022) demonstrated that clusters of responding AZs can be activated by Ca^2+^ transients. An active cluster (Figure 7A) was defined as two or more AZs in the immediate vicinity to each other, with closest neighbors being separated by no more than 1.5 μm, which produced optical events with the onsets separated by no more than 5 frames (200 ms). Notably, we found that numerous clusters of spontaneous release were activated upon HFS (Figure 7B). The unstimulated NMJs (Figure 7B, cyan) demonstrated mostly the clusters of two AZs, so that two next-door AZs produce events nearly simultaneously. Sometimes, clusters of three AZs were observed, and occasionally up to 6–8 AZs were included in a cluster. In contrast, the activity at the stimulated preparations (Figure 7B, magenta) showed the prominence of the clusters that included three AZs (note the mode of distribution at the value of AZs equal three). Furthermore, and sizes of the clusters frequently exceeded 10 AZs and sometimes were as large as 30–50 AZs, incorporating all the AZs within a synaptic bouton. This produced synaptic boutons with intense spontaneous activities that would last for seconds or even tens of seconds.

In summary, we found that a brief HFS tetanus activated numerous AZs for spontaneous activity, while more prolonged HFS tetanus converted AZs to their high-activity states and activated AZ clusters, thus generating hot spots of continuous asynchronous release.

## 4 Discussion

Our earlier study (Astacio et al., 2022) revealed the transient hot spots of spontaneous transmission, which likely represent the dedicated spontaneous communication cannel. The present study investigated how the hot spots of spontaneous transmission are regulated by HFS. We employed the HFS tetanus for either 2 or 60 s and found that the high activity states of individual AZs were significantly promoted by HFS, and this effect was most prominent for the prolonged tetanus (60 s). While the brief HFS tetanus (2 s) increased the spontaneous activity broadly across the entire AZ ensemble, the more prolonged tetanus (60 s) selectively activated the hot spots of spontaneous transmission.

It has long been recognized that HFS promotes synaptic transmission at both central and neuromuscular synapses (Atwood et al., 1989; Nicoll and Schmitz, 2005), however most of the studies focused on the enhancement in the evoked synaptic transmission. However, the recent realization that the evoked and spontaneous communication channels are at least partially segregated (Chanaday and Kavalali, 2018; Peled et al., 2014; Melom et al., 2013; Kavalali, 2018) raises the question of how the spontaneous communication channel is affected by HFS.

Initial studies at the *Drosophila* NMJ revealed that patterned brief HFS (four 1 s tetani at a 100 Hz frequency) induces elevated spontaneous activity (Yoshihara et al., 2005), which depends on retrograde signaling and presynaptic protein kinase A (PKA). A subsequent study (Cho et al., 2015) further investigated this mechanism and discovered that the HFS-induced spontaneous activity is associated with the PKA phosphorylation of the presynaptic protein complexin (Cpx). Notably, it was also shown (Astacio et al., 2022) that the hot spots of spontaneous transmission at rest depend on the protein fusion machinery, including the SNARE complex that mediates the attachment of SVs to the presynaptic membrane, and Cpx which associates with the SNARE complex. The present study demonstrated that these hot spots are selectively promoted by prolonged HFS, suggesting the HFS-induced Cpx phosphorylation as an underlying mechanism.

In addition, it was also shown that the activity at the hot spots of spontaneous transmission was promoted by Ca^2+^-dependent mechanisms, including opening of VGCCs and Ca^2+^ release from internal Ca^2+^ stores (Astacio et al., 2022). Thus, it is likely that both Ca^2+^-dependent and Ca^2+^-independent mechanisms contribute to the HFS-induced activation of hot spots of synaptic transmission.

Our understanding of the mechanisms controlling the hot spots of spontaneous transmission was assisted by the development of the three-state model which incorporated the high activity states of AZs, HA (active for minutes), as well as the states enabling sub-second bursts of events, SB. We found that the observed distributions of activities and latencies at individual AZs were well fit by the three-state model, including the distributions derived both at rest and upon HFS. Notably, at rest the HA states were predominantly influenced by the SNARE-Cpx fusion machinery, while the SB states depended on VGCC openings (Astacio et al., 2022). Importantly, the present study demonstrated that HFS promoted both HA and SB states of individual AZs, suggesting the coupling of Ca^2+^-dependent and Ca^2+^-independent mechanisms. In addition, the present study demonstrated that HFS activates the clusters of AZs, which were shown to depend on the internal Ca^2+^ stores (Astacio et al., 2022).

Thus, our results suggest that HFS promotes the hot spots of spontaneous activity via both Ca^2+^-dependent and Ca^2+^-independent mechanisms. One possibility is that HFS could generate local spots of Cpx deficiency or dysfunction. Indeed, it was demonstrated that Cpx deletion drastically elevates spontaneous activity (Huntwork and Littleton, 2007), and that HFS promotes Cpx phosphorylation leading to the enhancement of spontaneous transmission (Cho et al., 2015). Thus, it is a plausible hypothesis that HFS enhances the HA states of individual AZs by creating the focal spots lacking the active Cpx forms. In addition, we believe that this mechanism is enhanced by spontaneous openings of VGCCs (McCarthy and Kavalali, 2024; Oheim et al., 2006), which induce bursts of spontaneous fusion events, generating SB states at selected AZs. Finally, the miniature release of Ca^2+^ from internal Ca^2+^ stores (Rusakov, 2006) likely adds to the latter mechanism, transiently activating the clusters of AZs for spontaneous transmission.

What is the function of the activity-dependent hot spots of spontaneous transmission? The discovery that the synaptic growth in Drosophila is enhanced in the Cpx null mutant (Huntwork and Littleton, 2007), suggested a link between the spontaneous transmission and the neuronal outgrowth. A subsequent study (Cho et al., 2015) demonstrated a direct correlation between synaptic growth and spontaneous activity. In line with these findings, another study in Drosophila found that selectively disrupting spontaneous transmission inhibits the synaptic growth (Choi et al., 2014). Parallel studies at mammalian synapses demonstrated that the spontaneous release component is predominant in immature growing neurons, suggesting the pivotal role of spontaneous transmission in the developmental neuronal growth (Andreae and Burrone, 2018).

Notably, intense stimulation, including HFS, induces robust synaptogenesis that starts from the budding of new presynaptic boutons (Vasin et al., 2014; Ataman et al., 2008; Piccioli and Littleton, 2014). The boutons can be formed upon activity very rapidly, sometimes within minutes, while the postsynaptic specializations are formed subsequently within hours (Ataman et al., 2008; Piccioli and Littleton, 2014; Vasin et al., 2019).

The present study demonstrated that HFS generates the hot spots of spontaneous transmission, thus generating focal points for a positive feedback loop of activity. We hypothesize that these hot spots of spontaneous transmission can serve as tags for local neuronal outgrowth and synaptogenesis. Further experimentation is needed to test this hypothesis.

## Data Availability

The raw data supporting the conclusions of this article will be made available by the authors, without undue reservation.
